# Checks and Balances in Autoimmune Vasculitis

**DOI:** 10.3389/fimmu.2018.00315

**Published:** 2018-02-22

**Authors:** Rebeca Hid Cadena, Wayel H. Abdulahad, G. A. P. Hospers, T. T. Wind, Annemieke M. H. Boots, Peter Heeringa, Elisabeth Brouwer

**Affiliations:** ^1^Department of Pathology and Medical Biology, University of Groningen, University Medical Center Groningen, Groningen, Netherlands; ^2^Department of Rheumatology and Clinical Immunology, University of Groningen, University Medical Center Groningen, Groningen, Netherlands; ^3^Department of Medical Oncology, University of Groningen, University Medical Center Groningen, Groningen, Netherlands

**Keywords:** immune checkpoints, immune checkpoint inhibitors, immune-related adverse events, vasculitis, giant cell arteritis

## Abstract

Age-associated changes in the immune system including alterations in surface protein expression are thought to contribute to an increased susceptibility for autoimmune diseases. The balance between the expression of coinhibitory and costimulatory surface protein molecules, also known as immune checkpoint molecules, is crucial in fine-tuning the immune response and preventing autoimmunity. The activation of specific inhibitory signaling pathways allows cancer cells to evade recognition and destruction by the host immune system. The use of immune checkpoint inhibitors (ICIs) to treat cancer has proven to be effective producing durable antitumor responses in multiple cancer types. However, one of the disadvantages derived from the use of these agents is the appearance of inflammatory manifestations termed immune-related adverse events (irAEs). These irAEs are often relatively mild, but more severe irAEs have been reported as well including several forms of vasculitis. In this article, we argue that age-related changes in expression and function of immune checkpoint molecules lead to an unstable immune system, which is prone to tolerance failure and autoimmune vasculitis development. The topic is introduced by a case report from our hospital describing a melanoma patient treated with ICIs and who subsequently developed biopsy-proven giant cell arteritis. Following this case report, we present an in-depth review on the role of immune checkpoint pathways in the development and progression of autoimmune vasculitis and its relation with an aging immune system.

## Introduction

Age-associated changes in the immune system are thought to contribute to an increased susceptibility for autoimmune diseases. These changes include shifts in immune cell numbers, distribution, and function in conjunction with alterations in cell surface protein expression. One important class of surface proteins expressed on immune cells is immune checkpoint molecules, which regulate T cell activation by relaying positive (costimulatory) and negative (coinhibitory) signals. The balance between the expression of coinhibitory and costimulatory molecules is crucial in fine-tuning the immune response and preventing autoimmunity.

By exploiting the activation of specific inhibitory signaling pathways, cancer cells are able to evade recognition and destruction by the host immune system. Currently, several coinhibitory molecules are targeted by antibody-based antagonist biologicals in cancer immunotherapy. The rationale for this approach is that blockade of inhibitory checkpoints causes an unrestrained immune response allowing the host’s tumor-specific T cells to attack the tumor cells. This immune checkpoint blockade strategy has proven to be very effective, producing long-lasting antitumor responses in multiple cancer types (1–3).

Nevertheless, immune checkpoint therapy has its disadvantages. Blocking the inhibitory signaling pathways may unleash reactivity to healthy tissues, which consequently may result in inflammatory manifestations in patients receiving these agents, termed immune-related adverse events (irAEs) (3–6). These irAEs are often relatively mild, but more severe irAEs have been reported as well including several forms of vasculitis such as granulomatosis with polyangiitis (GPA) (7), lymphocytic vasculitis (8), and polymyalgia rheumatica/giant cell arteritis (9–11) (Table 1).

**Table 1:** Reported cases of vasculitis developed after immune checkpoint inhibitor treatment.

Reference	Reported case
van den Brom et al. (7)	Granulomatosis with polyangiitis induced by immune checkpoint inhibition (α-CTLA-4 and α-PD-1)
Minor et al. (8)	Lymphocytic vasculitis of the uterus in a patient with melanoma receiving ipilimumab
Goldstein et al. (9)	Polymyalgia rheumatica/giant cell arteritis occurring in two patients after treatment with ipilimumab
Hodi et al. (10)	Giant cell arteritis in a patient with metastatic melanoma receiving bevacizumab plus ipilimumab
Calabrese et al. (11)	Rheumatic immune-related adverse events of checkpoint therapy for cancer (PMR-like syndrome in three patients: two receiving α-PD-1 and one receiving α-CTLA-4 and α-PD-1)

However, little is known about the role of immune checkpoints in vasculitis. In this article, we discuss the evidence that age-associated changes in expression and function of immune checkpoint molecules leads to an imbalance of the immune system. An immune system out of balance is prone to tolerance failure and the development of autoimmune vasculitis. The topic is introduced by a case report from our hospital describing a melanoma patient treated with immune checkpoint inhibitors (ICIs) and who subsequently developed biopsy-proven giant cell arteritis. This case study sets the stage for a more in-depth review on the role of immune checkpoint pathways in the development and progression of autoimmune vasculitis and its relation with the aging immune system.

## Case Vignette

A 70-year-old man with a history of hepatitis A and who had a myocardial infarction in 2001 developed a melanoma of the skin of the left temple in 2015. He was diagnosed with stage IIIB BRAF mutated melanoma and was treated with modified radical dissections including a parotidectomy, a neck dissection, and a free skin transplantation on June 8, 2015.

In August 2015, he started with adjuvant treatment in a double-blind study CA209-238 (Efficacy Study of Nivolumab Compared to Ipilimumab in Prevention of Recurrence of Melanoma after Complete Resection of Stage IIIb/c or Stage IV Melanoma (CheckMate 238); ClinicalTrials.gov number, NCT02388906) until April 2016. In April 2016, he was referred to the rheumatology and clinical immunology department with the following complaints: fatigue, low-grade fever with a temperature reaching 38.5 C, night sweats, and weight loss of 4 kg in 2 weeks. He had also experienced continuous pain for 4 weeks in his jaws and mastoid muscles. The right temple and masseter muscle were painful upon palpation, and his pain increased upon chewing. He had no hair pain or visual problems. He developed also new-onset pain and stiffness in his upper legs, neck, and shoulders. He had no pain or stiffness in his smaller joints, excluding a diagnosis fitting with arthritis.

On physical examination, he was fatigued, his blood pressure was 140/70 (upon measurement in both arms), his height was 1.78 m, and his weight 62 kg. His right temporal artery was painful, and his left temporal artery was not palpable (status after radical surgery). His shoulders and upper legs were painful upon movement. He had no infectious, gastrointestinal, or skin symptoms. His blood tests showed an elevated ESR of 93 mm/h, CRP of 52 mg/L, a hemoglobin level of 7.8 mmol/L (in October 2015, before immune checkpoint treatment, these values were ESR of 37 mm/h, CRP of 1.6 mg/L, and a hemoglobin level of 8.1 mmol/L).

An ultrasound of the temporal and axillary arteries, a PET/CT scan, and a temporal artery biopsy were performed. No halo fitting with GCA was observed upon US of his temporal and axillary arteries and muscles. The PET/CT did not show signs of large vessel vasculitis (LVV), myositis, infections, or metastasis, but did show some uptake surrounding both hips that would fit with a diagnosis of PMR. An additional MRI was performed, which did not show cerebral or leptomeningeal metastasis, and the masseter and temporal muscle and temporal and facial artery on the right side appeared to be normal. The ophthalmologist and the neurologist found no signs and symptoms that would fit the diagnosis of GCA and also ruled out trigeminal neuralgia.

**Figure 1:**
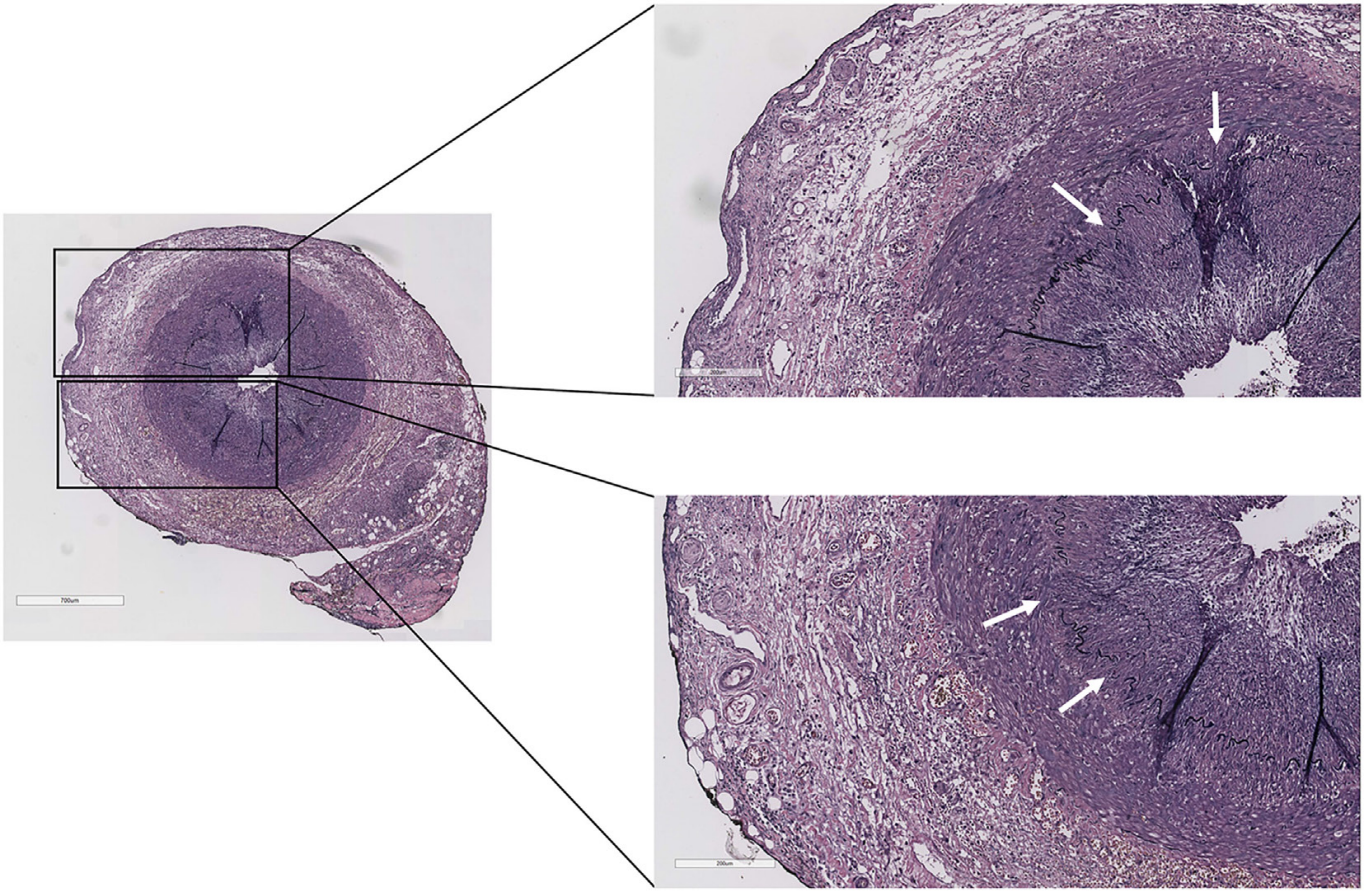
Temporal artery biopsy of the case report patient showing transmural inflammation of the adventitial, medial, and intimal layers with a fragmented internal and external lamina elastic (white arrows) (Verhoeff–Van Gieson staining).

The complaints of the patient were progressive, and his ESR and CRP remained high, while his right temporal artery increased in size and remained painful upon palpation. On May 23, 2016, the patient underwent a temporal artery biopsy from his right temporal artery, which revealed a transmural inflammation of the adventitial, medial, and intimal layers of the temporal artery with a fragmented internal and external lamina elastic, diagnostic for GCA (Figure 1). On May 24, the patient started with high-dose prednisolone (60 mg/day), which was tapered to 30 mg/day on May 25 (due to severe side effects) and gradually tapered to 2.5 mg/day on November 3, 2016. Disease activity of GCA was monitored according to the BSR definition that a disease relapse should be suspected in patients with a return of symptoms of GCA, ischemic complications, or unexplained constitutional or polymyalgic symptoms. (Relapse is usually associated with an increase in erythrocyte sedimentation rate/C-reactive protein, but may occur with normal inflammatory markers.)

Unfortunately, in October 2016, he had developed metastasized melanoma (lymph nodes and lung), and his previous adjuvant treatment was deblinded (not allowed to mention in this article nivolumab or ipilimumab as the study is not yet deblinded). On November 3, he started with a different checkpoint inhibitor. He had some persistent smoldering low-grade GCA complaints, which increased on this treatment. The complaints consisted of a headache on his left side and pain and stiffness in his neck and upper legs, and he had a painful temporal artery on his left side. The ESR of 37 mm/h and CRP of 7 mg/dL were slightly increased, suggesting a GCA relapse. The prednisolone dose was increased to 10 mg/day. Infusions with checkpoint inhibition were continued, and he was advised to take an increased prednisolone dose of 20 mg at day 2 and 3 after these infusions.

**Figure 2:**
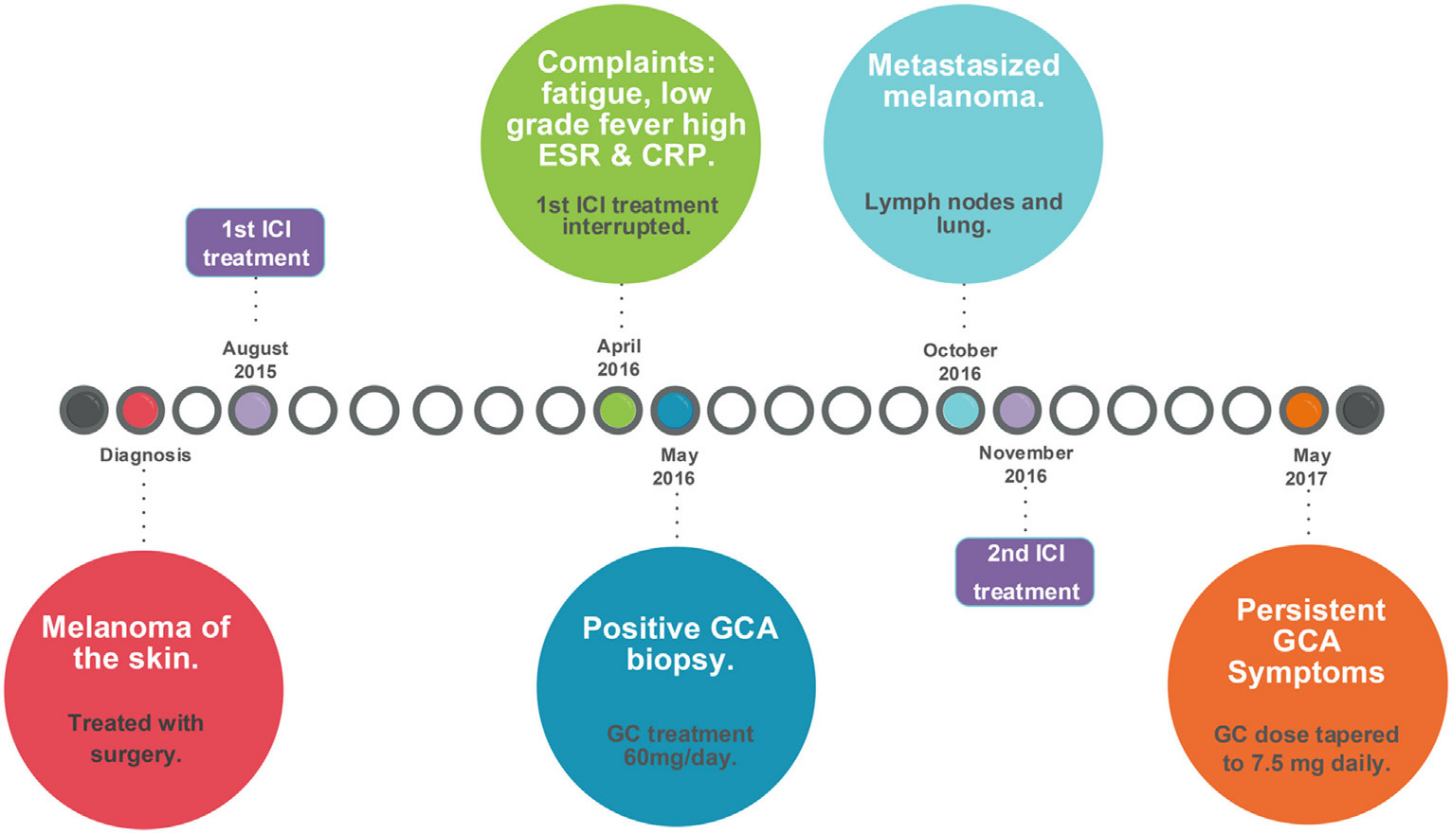
Timeline of events leading to the development of GCA induced by checkpoint immunotherapy. CRP, C-reactive protein; ESR, erythrocyte sedimentation rate; GC, glucocorticoids; GCA, giant cell arteritis; ICI, immune checkpoint inhibitor. Surgery included a modified radical dissections including a parotidectomy, a neck dissection, and a free skin transplantation on June 8, 2015, for stage IIIb melanoma, which was followed by inclusion in the CA209-238 study [Efficacy Study of Nivolumab Compared to Ipilimumab in Prevention of Recurrence of Melanoma After Complete Resection of Stage IIIb/c or Stage IV Melanoma (CheckMate 238); ClinicalTrials.gov number, NCT02388906].

In May 2017, he still had signs and symptoms that fit with active GCA, especially jaw complaints upon chewing but no headache. The ESR was 4 mm/h and CRP was <0.3 mg/dL. He was advised to taper the prednisone to 7.5 mg/day to control the GCA without giving too much immunosuppression. A schematic representation of GCA development induced by immune checkpoint blockade is given in Figure 2.

**Figure 3:**
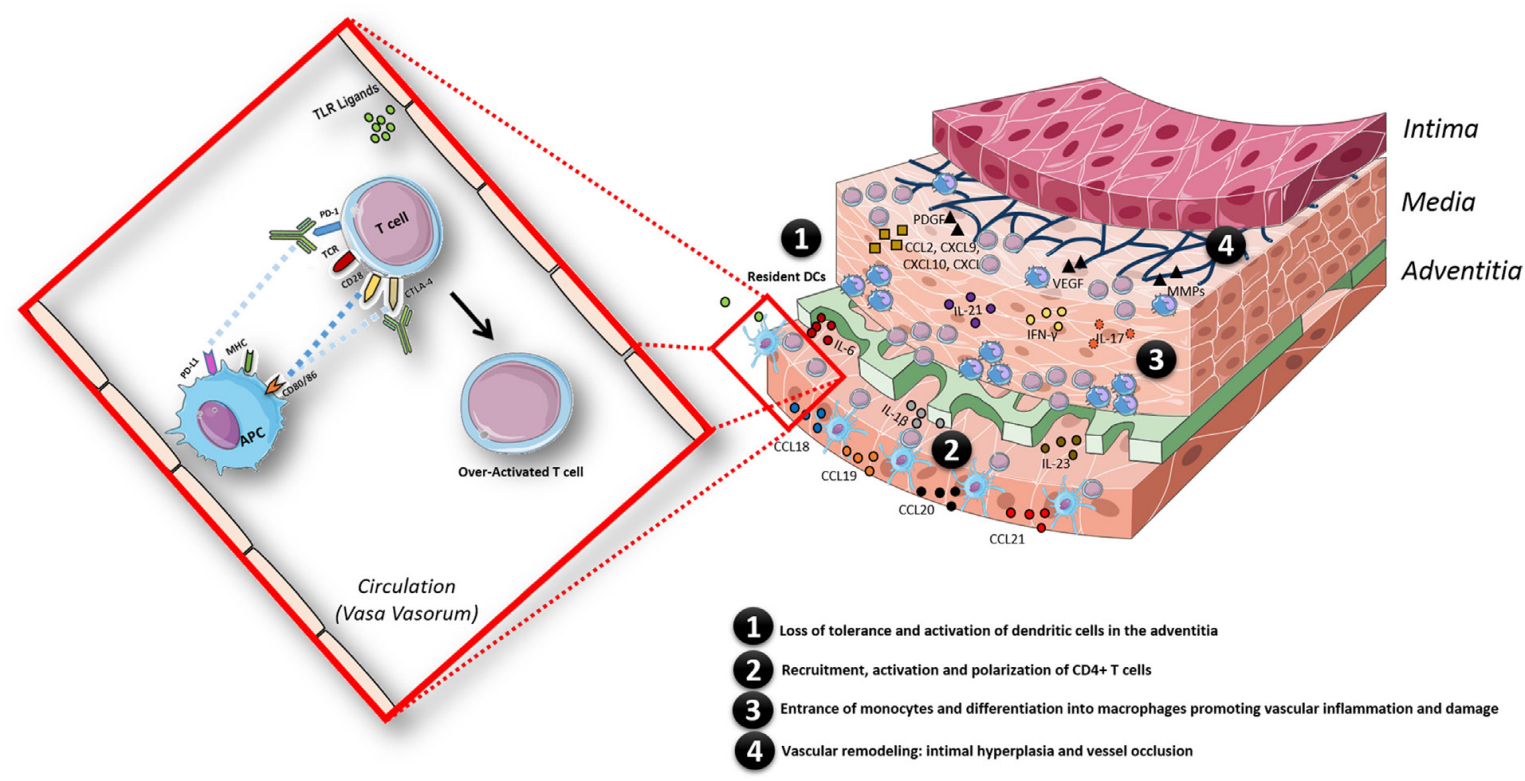
Schematic model of the pathogenesis of giant cell arteritis, facilitated by the state of chronic inflammation in aged individuals and in addition by an overactivated immune system triggered by immune checkpoint inhibitor treatment. The inflammatory response in the arterial wall is initiated when resident dendritic cells (DCs) sense danger signals *via* pattern recognition receptors such as toll-like receptors. Activated DCs produce chemokines (CCL18, CCL19, CCL20, and CCL21), which recruit CD4+ T cells; once recruited in the arterial wall, CD4+ T cells are activated by DCs presenting still undefined antigen(s). The presence of pro-inflammatory cytokines (IL-6, IL-1β, IL-23, IL-18, and IL-12) in the microenvironment polarizes CD4+ T cells toward Th1 and Th17 cells, which produce large amounts of IFN-γ and IL-17. Eventually, monocytes enter the vascular wall and differentiate into macrophages promoting vascular inflammation by secreting cytokines and vascular damage *via* secretion of matrix metalloproteinases (MMPs). Macrophages, giant cells or injured VSMC also produce growth factors such as platelet-derived growth factor (PDGF) and vascular endothelial growth factor (VEGF). This results in vascular remodeling: intimal hyperplasia and vessel occlusion. The whole process is facilitated by a state of chronic inflammation as observed in aged individuals and additionally by an overactivated immune system triggered by immune checkpoint therapy treatment in this case.

The case described above is a prime example of an adverse consequence upon immune checkpoint therapy, illustrating that removing the natural brakes of the immune system may lead to a breach of tolerance and development of autoimmunity, such as LVV in this example (Figure 3). In this case, the patient was treated with in total two ICIs. ICIs are FDA-approved drugs in the treatment of advanced melanoma. Ipilimumab was the first checkpoint inhibitor approved by the FDA in 2011 for the treatment of advanced melanoma (12), and it showed improved efficacy and survival benefits compared to other chemotherapeutic agents (13). PD-1 inhibition with pembrolizumab and nivolumab also has proven to be effective in advanced melanoma (14–17) and was approved by the FDA in 2014.

Besides anti-PD-1 agents, the FDA has also recently approved antiprogrammed death-ligand 1 (PD-L1) agents for the treatment of patients with several types of cancer (18, 19). In the coming years, the approval of new ICIs or a combination of checkpoint-targeting agents that are currently under investigation in oncology clinical trials is expected. Approval of these drugs will translate into an increased use of immunotherapies, prompting the investigation of the underlying mechanisms of immune checkpoint regulation to avoid unwanted adverse events such as the one presented in the case above.

Although there is an increased awareness of the more common irAEs upon immune checkpoint therapies, rare but severe and potentially life-threatening autoimmune manifestations, such as vasculitis, should be taken into account when evaluating the benefit of tumor destruction and the associated risks of immunotoxicity. Some of the toxicities related to immune checkpoint therapy reported in multiple studies are summarized in Table 2 (16, 20, 21). The reported rate for the more common irAEs, which involve the skin, gastrointestinal system, and endocrine system are comparable when using only one ICI, but the reported rate for these irAEs significantly increases when a combination of therapies is used. For those types of disorders that are not as common, the reporting rate is very low, even when combination therapy is used. The frequency of autoimmune complications may be underestimated due to the fact that follow-up in clinical trials is usually short, and the development of autoimmune toxicities can have a delayed onset (22).

**Table 2:** Frequency of selected immune-related adverse events associated with immune checkpoint inhibitor treatment.^a^

Type of disorder	Anti-CTLA-4 (ipilimumab)	Anti-PD-1 (nivolumab or pembrolizumab)	Combination therapy
**Skin (rash)**			
Robert et al. (16)^b^	14.5	14.7	NR
Postow et al. (20, 37)^c^	26	NR	41
Larkin et al. (21)^d^	32.8	25.9	40.3
Range	15–33%	15–26%	40–41%
**Gastrointestinal (diarrhea)**			
Robert et al. (16)^b^	22.7	16.9	NR
Postow et al. (20, 37)^c^	37	NR	45
Larkin et al. (21)^d^	33.1	19.2	44.1
Range	23–37%	17–19%	44–45%
**Endocrine (hypothyroidism)**			
Robert et al. (16)^b^	2	10.1	NR
Postow et al. (20, 37)^c^	15	NR	16
Larkin et al. (21)^d^	4.2	8.6	15
Range	2–15%	9–10%	15–16%
**Others (arthralgia)**			
Robert et al. (16)^b^	5.1	9.4	NR
Postow et al. (20, 37)^c^	9	NR	11
Larkin et al. (21)^d^	6.1	7.7	10.5
Range	5–9%	8–9%	10–11%

*^a^Values are the percentage of treated patients who experienced adverse events of any grade (based on the common terminology criteria for adverse events grading system)*.

*^b^Ipilimumab (*N* = 256), anti-PD-1 agent used: pembrolizumab (*N* = 278)*.

*^c^Ipilimumab (*N* = 46); combination therapy used: nivolumab plus ipilimumab (*N* = 94)*.

*^d^Ipilimumab (*N* = 311); anti-PD-1 agent used: nivolumab (*N* = 313); combination therapy used: nivolumab plus ipilimumab (*N* = 313)*.

*NR, not reported*.

To better understand the mechanisms of action of ICIs and the adverse consequences derived from their use, it is essential to consider the various immune functions that these checkpoints control; this issue is addressed in the following sections.

## Coinhibitory Checkpoint Pathways

The two inhibitory checkpoint pathways that have been most widely studied in oncology are the CTLA-4 and PD-1 pathways. Immune responses are negatively regulated by these pathways at different levels and by different mechanisms.

### CTLA-4 Pathway

The ability of the immune system to protect from harm and prevent unnecessary tissue injury is maintained by a delicate balance between costimulatory and coinhibitory molecules. One example of this delicate balance is the interaction between the coinhibitory molecule CTLA-4 and its counterpart, the costimulatory molecule CD28. Both CD28 and CTLA-4 are expressed on T cells and control the early stages of T cell activation (23–25). Once antigen recognition occurs through engagement of the T cell receptor (TCR) with the cognate antigen–MHC complex, presented by antigen-presenting cells (APCs), CD28 binds to CD80 and CD86; this binding strongly amplifies TCR signaling to activate T cells (25–28). Within 48 h of activation, expression of CTLA-4 is upregulated on activated T cells (3). As CD28 and CTLA-4 share identical ligands, the latter dampens T cell activation by outcompeting the former in binding to CD80 and CD86 (24, 29–31). CTLA-4 can further decrease activation by sending a signal to APCs to reduce CD80/86 expression (32) and secrete indoleamine 2,3-dioxygenase (IDO), an enzyme that catalyzes tryptophan degradation (33), disabling T lymphocytes to proliferate due to tryptophan shortage (34). Activated CD8+ T cells also express CTLA-4, which suppresses helper T cell activity and enhances the immunosuppressive activity of regulatory T (Treg) cells (35). Treg cells constitutively express CTLA-4, which on the one hand leads to Treg cell proliferation and enhanced production of IL-35, IL-10, TGF-β, and IDO. On the other hand, on effector T (Teff) cells, CTLA-4 engagement causes a decreased activation and proliferation (6, 36).

Collectively, as CTLA-4 regulation takes place early in the process of T cell activation and augments Treg function, it is likely that its blockade leads to an unrestrained non-specific activation of the immune response. This broad activation may explain the wide variety of adverse events seen when this pathway is blocked (25, 37).

### PD-1 Pathway

Although both CTLA-4 and PD-1 are negative checkpoints, PD-1 exerts its function at different levels and *via* different mechanisms. Upon engagement to either PD-L1 (also known as CD274 and B7-H1) or programmed death-ligand 2 (PD-L2; also known as CD273 and B7-DC), tyrosine phosphorylation of the PD-1 cytoplasmic domain occurs and tyrosine phosphatase SHP-2 is recruited, resulting in disruption of the TCR signaling cascade (38–41). These effects ultimately block T cell proliferation, diminish cytokine production and cytolytic function, and impair T cell survival (3, 42, 43). The cellular expression of PD-1 is broader than that of CTLA-4; for example, B cells and natural killer cells also express and upregulate PD-1 upon activation (25, 44), thereby temporarily dampening their effector functions (39). Another important subset of T cells that highly expresses PD-1 is Treg cells, and it has been demonstrated that PD-1 ligation on these cells enhances their immunosuppressive activity (43, 45). Both the PD-L1 and PD-L2 ligands are expressed on APCs and other hematopoietic and non-hematopoietic cell types (46).

In preclinical models, PD-1/PD-L1 pathway inhibition also generates antitumor activity and enhances autoimmunity (47). However, the autoimmune phenotypes of mice with PD-1 or CTLA-4 deficiencies are different. CTLA-4 deficiency results in a more severe, non-specific autoimmune phenotype as it affects both cell-intrinsic activities (on Teff cells) and cell-extrinsic activities (on Treg cells) (48). By contrast, PD-1 deficiency results in a mild and chronic autoimmune phenotype since it is mainly manifested as cell-intrinsic alterations of Teff cells (3, 48). Since PD-1 activation suppresses the immune response during the effector phase of T cell activation and upon repeated antigen exposure, PD-1 blockade probably targets a more restricted assortment of T cells than CTLA-4 blockade (3).

## Lessons Learned from Oncology

The cancer immunity cycle described by Chen and Mellman in 2013 has become a useful framework for immunotherapy research. Briefly, the authors refer to seven steps, which need to be initiated and allowed to proceed and expand iteratively for an anticancer immune response to effectively kill cancer cells. These steps involve: step 1: the release of cancer antigens, step 2: presentation of those antigens through APCs and dendritic cells (DCs), step 3: T cell priming and activation within the lymph node, step 4: T cell trafficking to tumors, step 5: T cell infiltration into the tumor, step 6: recognition of cancer cells by T cells, and finally, step 7: cancer cell killing, which restarts the cycle (49). In each step described above, as in all of the immune system processes, checks and balances are required to perform optimally, which in cancer patients are ablated due to cancer’s many strategies to evade recognition by the host immune system. Obstacles encountered in one or several steps of the cancer-immunity cycle are the target of immunotherapy; therefore, combination of approaches with therapies stimulating various and different steps of the cycle may result in higher response rates (50) and consequently more irAEs.

### Effect of Immunotherapies on Checkpoint Molecule Expression and Function

In cancer patients, anti-CTLA-4 treatment lowers the threshold required for T cell activation, which leads to an expansion of circulating low-avidity T cells (51), resulting in a sustained immune response. In addition, it has been shown that anti-CTLA-4 therapy promotes antitumor activity by a selective reduction of intratumoral Treg *via* Fc-γR-mediated depletion (52), impairing Treg cell survival and function along with concomitant activation of Teff cells (35, 53). In addition, Th17 cells, which are implicated in many autoimmune and chronic inflammatory disorders (54) and in tumor eradication (55) processes, are also affected by CTLA-4 blocking. In cancer patients, it has been demonstrated that upon anti-CTLA-4 treatment, the number of circulating Th17 cells in patients increases, especially in those patients who developed clinically relevant inflammatory and autoimmune toxicities (56).

Recently, Wei et al. confirmed that distinct cellular mechanisms underlie anti-CTLA-4 and anti-PD-1 checkpoint blockade. The authors concluded that both checkpoint blockade therapies targeted only specific tumor-infiltrating exhausted-like CD8 T cells and that the effect of these agents primarily differed in the expansion of inducible costimulator (ICOS) + Th1-like CD4 effector cells induced by the anti-CTLA-4 agent (57). Furthermore, additional studies in cancer patients show that after targeting CTLA-4 with ipilimumab, responding patients have increased ICOS + T cells (58, 59). Several research groups have reported that there appears to be a compensatory upregulation of alternative checkpoints following immune checkpoint blockade (60–62). Very recently, a study by Gao et al. demonstrated that the inhibitory immune checkpoint molecules PD-L1 and V-domain Ig suppressor of T cell activation are both upregulated in CD4+ and CD8+ T cells and CD68+ macrophages of prostate cancer patients in response to ipilimumab therapy (62). The upregulation of alternative checkpoints as a compensatory mechanism might explain the lack of response or partial tumor regression observed in preclinical models (60, 61) and in cancer patients when treated with anti-CTLA-4 or anti-PD-1 monotherapy (16, 62, 63).

Such compensatory mechanism by which the immune system strives toward balance is supported by increasing evidence, indicating that basic signaling mechanisms of several immune checkpoint pathways are intertwined with each other forming a complex network that regulates the immune response. Kamphorst et al. found that CD28 signaling is essential for T cells to effectively respond to PD-1 blockade during chronic viral infection (64). Through conditional gene deletion, they showed a cell-intrinsic requirement of CD28 for CD8 T cell proliferation after PD-1 therapy (64). Moreover, Hui et al. reported that CD28 is strongly preferred over the TCR as a target for dephosphorylation by PD-1-recruited SHP-2 phosphatase, revealing that signaling through PD-1 occurs mainly by inactivating CD28 signaling (65). These data suggest that there is a broader interaction between PD-1 and CD28 than previously assumed, and such interaction might serve as a general mechanism for enhancing normal T cell responses and revitalizing exhausted T cells (66).

The unprecedented clinical success of cancer immunotherapy and the subsequent development of irAEs seen with these therapies have enabled researchers to study the underlying mechanisms of the early stages of autoimmunity. The expression of inhibitory receptors has been reported to be altered in many autoimmune diseases (67, 68), which suggests that signaling by inhibitory receptors is involved in the etiology of autoimmune diseases (67, 69). However, whether defective expression and/or function of immune checkpoints is a cause or consequence of autoimmunity and the ensuing autoimmune diseases is largely unknown. One factor that may be involved is age since aging is known to alter many aspects of the immune system and increases the susceptibility for the development of autoimmune diseases.

## Impact of Aging and Immunosenescence on Checkpoint Molecule Expression

As a result of aging-related changes in the immune system, the human body becomes more susceptible for developing cancer, autoimmune diseases, infections, and cardiovascular diseases (70–73). Aging impacts both the innate and adaptive constituents of the immune system, which lead to a dysregulated immune and inflammatory response contributing to the increased incidence of chronic immune-mediated diseases in elderly individuals (74).

The immune system of aged people shows an accumulation in the frequency of highly differentiated T cells of which, due to a greater homeostatic stability, CD4+ T cells are being less affected by the age-associated phenotypic and functional changes than CD8+ T cells (75, 76). These changes include loss of the cell surface costimulatory molecules CD27 and CD28, CD8+ T cells losing CD28 first followed by CD27 and vice versa for CD4+ T cells (77). Loss of the costimulatory molecule CD28 is a hallmark of the age-related decline of T cell function, which has been associated with a less-efficient capability to mediate immune responses in old individuals (78).

In addition to the loss of costimulatory molecules, there is an increase in the expression of inhibitory receptors, which adds to T cell dysfunction during aging (79). The expression of the inhibitory checkpoint molecule, CTLA-4 increases with age (80), whereas the expression of PD-1 is considered to be dependent on viral status rather than age and may also serve as a useful marker on viral-specific CD8+ T cells to indicate the degree of T cell exhaustion (41). In chronic viral infections and tumor microenvironments, PD-1-expressing exhausted cells lose their ability to produce IFN-γ and TNF-α and therefore become dysfunctional (81–83).

The age-related changes and deterioration of the immune system have been linked to immunosenescence (84), a term referring to the continuous remodeling of lymphoid organs, which leads to reduced immune function in elderly people (85). One of the major factors that fuels immunosenescence appears to be the lifelong chronic antigen load (86, 87) including leakage of microbial products from the gut to the circulation, resulting in continuous stimulation of both innate and adaptive immunity. Altogether, these changes lead to a chronic pro-inflammatory state favoring the development of age-associated (auto) inflammatory diseases (88).

## Role of Immune Checkpoints in the Development of Immune-Mediated Vasculitis

Vasculitides are a heterogeneous group of inflammatory disorders characterized by inflammation of the blood vessel wall. The clinical manifestations are determined by the localization, the type of vessel involved, and the nature of the inflammatory process (89). The Chapel Hill nomenclature classifies non-infectious vasculitides mainly according to the type of vessel affected: LVV, medium vessel vasculitis (MVV), and small vessel vasculitis (SVV). LVV affects the aorta and its main branches, and the primary vasculitides in this group are GCA and Takayasu arteritis. MVV affects the main visceral arteries and its branches; examples of diseases in this group are polyarteritis nodosa and Kawasaki disease. Finally, SVV is further subdivided into antineutrophil cytoplasmic antibody (ANCA)-associated vasculitis (AAV) and immune complex SVV. The major clinicopathologic variants of AAV are GPA, microscopic polyangiitis (MPA), and eosinophilic granulomatosis with polyangiitis (EGPA) (90).

Antineutrophil cytoplasmic antibody-associated vasculitis is predominantly disease of the elderly. The incidence of AAV increases with age, peaking in those aged 65–74 years (91–93). A hallmark of the AAV is the presence of autoantibodies directed at neutrophil cytoplasmic constituents (ANCA) (94, 95). The target antigens of ANCA in the AAV are proteinase 3 (PR3) and myeloperoxidase (MPO) where GPA is primarily associated with PR3-ANCA and MPA and EGPA with MPO-ANCA. The immunopathological model of AAV in the acute effector phase is centered around ANCA and pro-inflammatory stimuli, most likely of infectious origin, which synergize in initiating a destructive inflammatory process (94, 95). A central event in this process is ANCA-mediated neutrophil activation resulting in the generation of reactive oxygen species (ROS), degranulation and cytokine production, a process that is greatly facilitated by minor (pro-)inflammatory stimuli that prime the neutrophil to interact with ANCA. Upon disease progression, acute vasculitis lesion transform into lesions that predominantly contain macrophages and T cells.

Although data on checkpoint expression in AAV patients are scarce, Wilde et al. reported increased expression of PD-1 on circulating T helper cells of GPA patients, whereas T cells in renal lesions mostly lacked PD-1 (96). The authors found that PD-1 expression was positively correlated with expansion of memory T cells, CD28^null^ T cells, as well as with T cell activation. In addition, PD-1 expression was found to be enhanced on pro-inflammatory IFN-γ T cells in GPA patients. These observations suggested that increased PD-1 expression on T cells might counterbalance persistent T cell activation (96).

Furthermore, Slot et al. analyzed single-nucleotide polymorphisms in the genes encoding PD-1 and CTLA-4 describing SNP frequencies in GPA patients that could explain hyperreactivity of T cells in these patients (97). Interestingly, in 2016, our group reported for the first time the development of GPA after sequential immune checkpoint inhibition with anti-CTLA-4 and anti-PD-1 treatment, as well as the first report of vasculitis observed after anti-PD-1 treatment (7). In that case report, we hypothesized that anti-CTLA-4 treatment induced PR3-ANCA production, which created the conditions necessary for the development of GPA, a process that was rapidly amplified by anti-PD-1 treatment (7).

GCA, the most common vasculitis after 50 years of age (98, 99), is thought to be caused by both changes in the aging vessel wall and in the immune system. The immunopathological model of GCA can be divided into four phases: in phase 1, there is a loss of tolerance (cause unknown) and activation of resident DCs of the adventitia, which results in the recruitment, activation, and polarization of CD4+ T cells (phase 2). Once recruited and activated in the arterial wall, the presence of pro-inflammatory cytokines (e.g., IL-12, IL-18, IL-23, IL-6, and IL-1β) in the microenvironment polarizes CD4+ T cells toward Th1 and Th17 cells. Th1 and Th17 are responsible for the production of large amounts of IFN-γ and IL-17, respectively, which ultimately leads to the recruitment of CD8+ T cells and monocytes (phase 3). Vascular remodeling (phase 4) starts when the IFN-γ-stimulated monocytes differentiate into macrophages and vascular smooth muscle cells differentiate into myofibroblasts producing IL-6, IL-1β, TNF-α, and vascular endothelial growth factor (99). This amplifies the local inflammatory response causing the release of toxic mediators for the arterial tissue such as ROS and matrix metalloproteinase, which eventually results in remodeling processes leading to intima proliferation and vascular occlusion (99, 100).

Accumulating evidence, including the case herein reported, points to an important role of immune checkpoints in the development of GCA. This is also emphasized by the demonstrated efficacy of abatacept; a new treatment for GCA (99, 101). This agent is a soluble fusion protein consisting of the ligand-binding domain of CTLA-4 and the Fc region derived from IgG1. CTLA-4-Ig binds to the APC B7 (CD80/86) molecule, thereby blocking B7 interaction with the CD28/CTLA-4 receptor on the T cell (102). By contrast, ipilimumab antagonizes the action of CTLA-4, thus enhancing immune reactivity by releasing this immunosuppressive checkpoint.

The involvement of immune checkpoints in the development of autoimmune side events is further supported by evidence from oncology, which shows that both CTLA-4 and PD-1 blockade result in enhanced Th17 cell responses and impaired Treg survival and function (52, 53, 56, 103). In addition, PD-1 blockade results in enhanced Th1 cell responses and increased production of cytokines such as IL-6 and IL-17 (103). This T cell functional flexibility and plasticity might be one of the mechanisms involved in the induction of autoimmune side effects (6).

In addition to CTLA-4 involvement in GCA, a recent study indicates that the immunoprotective PD-1/PD-L1 signaling pathway is affected as well. The study showed that tissue-residing DCs of GCA patients were low in PD-L1, whereas the majority of vasculitic T cells at the site of inflammation expressed PD-1 (104). Moreover, the *in vivo* vasculitogenic potential of PD-1 blockade was demonstrated using a humanized mouse model system of vasculitis, the Human Artery-Severe Combined Immunodeficiency Mouse Chimera model. Briefly, human axillary arteries were engrafted into NSG mice, and PBMCs from GCA patients or healthy individuals were adoptively transferred into the chimeras; chimeras were randomly assigned to treatment with PD-1 antibody or isotype control antibody. In this model, the authors confirmed that inhibiting PD-1/PD-L1 interaction enhanced tissue inflammation as GCA PBMCs but not healthy PBMCs were able to induce vasculitis. More specifically, PD-1 blockade enabled very few healthy T cells to enter the vascular wall, while PBMCs from GCA patients induced vessel wall inflammation. These observations suggested that T cells from GCA patients are especially vulnerable to PD-1 blockade (104, 105).

Zhang et al. demonstrated that in GCA a breakdown in PD-1/PD-L1 checkpoint resulted in unleashed vasculitic immunity and that such breakdown was responsible for the pathogenic remodeling of the inflamed arterial wall (104). The authors reported that PD-1 blockade gave rise to T cells producing IFN-γ, IL-17, and IL-21, which sustained multifunctional effector functions associated with the rapid outgrowth of hyperplastic intima and the induction of microvascular neoangiogenesis (104). Worthy of note, T cells producing IFN-γ, IL-17, and IL-21 play an important role in GCA and contribute to the pathogenesis of the disease (106, 107). Furthermore, PD-1 blockade biased T cells toward increased T-bet and RORC expression and diminished FoxP3 expression (104).

## Concluding Remarks

During the past decade, the introduction of ICIs has revolutionized cancer therapy and has proven to be a very effective strategy in inducing durable antitumor responses in multiple cancer types. Increasing evidence supports the idea that immune checkpoints cannot be regarded as separate pathways but as a complex network functioning in concert to maintain the delicate balance in the immune system. However, despite the clear therapeutic benefit, it is undeniable that the induction of irAEs is a serious disadvantage. It has become clear that data on safety of immune checkpoint therapies need further study in elderly individuals (85). It might be that the patient’s age is a relevant risk factor for irAEs (108) as the immune system of an elderly person is likely to demonstrate age-associated changes in checkpoint expression and function, which may be altered due to the chronic, low-grade inflammation. These changes imply that elderly patients will respond differently to ICI therapy than do younger patients evaluated in clinical trials.

Collectively, age-related changes and alterations in signaling pathways are complex and interconnected. These changes are likely to influence DC, Teff, and Treg pathways, increasing the likelihood of T cell suppression in the elderly (79). Indeed more research is needed to understand the link between age-related cellular and molecular changes and their potential influence on DC and T cell pathways leading to the development of autoimmunity. Nonetheless, lessons learned from the oncology field are valuable, enabling researchers to realize that the immune system is capable of reconfiguring the immune checkpoint complex network after modulation using ICIs. The altered expression of inhibitory receptors as seen in vasculitis patients, such as the abnormalities in the PD-1/PD-L1 pathway (105), hints at the involvement of immune checkpoints in disease development. Perhaps the use of agonistic inhibitory checkpoint molecules to halt self-damaging responses could restore the checks and balances, which are reported to be deficient in vasculitis.

## Ethics Statement

Written informed consent was obtained from the patient prior to presenting the case.

## Author Contributions

All authors listed have made a substantial, direct, and intellectual contribution to the work and approved it for publication.

## Conflict of Interest Statement

The authors declare that the research was conducted in the absence of any commercial or financial relationships that could be construed as a potential conflict of interest.
